# Supplementary material for: The r’-Wave Algorithm: A New Diagnostic Tool to Predict the Diagnosis of Brugada Syndrome after a Sodium Channel Blocker Provocation Test
Source: Sensors (Basel). 2023 Mar 16;23(6):3159. doi: 10.3390/s23063159 (PMC10056571; doi:10.3390/s23063159)
Supplement: Supplementary file 1 [file sensors-23-03159-s001.zip › sensors-2272275-supplementary.pdf]

SUPPLEMENTAL MATERIALS

The r'-Wave Algorithm: A New Diagnostic Tool to Predict the Diagnosis of Brugada Syndrome at Sodium Channel Blocker Provocation Test

Supplemental Tables

Supplemental Table S1

Supplemental Table S2

Supplemental Table S3

Supplemental Table S1. Cut-off values of the r'-wave algorithm in the Test cohort

Cut-off	Sensitivity	Specificity	Youden Index
≥1	100.0%	48.6%	0.486
≥2	100.0%	76.7%	0.767
≥3	83.2%	80.0%	0.632
≥4	71.9%	91.4%	0.633

Supplemental Table S2. Cut-off values of the r'-wave algorithm in the Validation cohort

Cut-off	Sensitivity	Specificity	Youden Index
≥1	96.9%	40.0%	0.369
≥2	90.0%	83.0%	0.73
≥3	75.8%	91.4%	0.672
≥4	51.5%	100.0%	0.515

Supplemental Table S3. Diagnostic characteristics of β-angle, α-angle, DBT- 5mm, DBT- iso and triangle base/height ratio cut-off values in studies.

	Cut-off	Article	Sensitivity	Specificity
β-angle	≥23°	van der Ree et al.[6]	77%	62%
	≥23°	Ohkubo et al.[7]	100%	54%
	≥23°	Vetta et al.[16]	83%	65%
	≥23°	Validation cohort	97%	23%
	≥36.8°	van der Ree et al.[6]	41%	98%
	≥36.8°	Serra et al.[5]	86%	95%
	≥36.8°	Validation cohort	85%	54%
	≥38.6°	van der Ree et al.[6]	41%	100%
	≥38.6°	Serra et al.[5]	85%	96%
	≥38.6°	Validation cohort	76%	77%
	≥58°	Ohkubo et al.[7]	23%	100%
	≥58°	van der Ree et al.[6]	17%	100%
	≥58°	Vetta et al.[16]	35%	98%
	≥58°	Chevallier et al.[4]	79%	83%
	≥58°	Gottshalk et al.[15]	60%	78%
	≥58°	Validation cohort	42%	100%
DBT- 5mm	≥160 ms	Serra et al.[5]	85%	96%
	≥160 ms	Validation cohort	61%	77%
	≥160 ms	Gottshalk et al.[15]	80%	40%
α-angle	≥50°	Chevallier et al.[4]	71%	79%
	≥50°	Validation cohort	22%	100%
DBT- iso	≥60 ms	Serra et al.[5]	95%	78%
	≥60 ms	Validation cohort	88%	31%
Triangle base/height ratio	≥0.8	Serra et al.[5]	82%	92%
	≥0.8	Validation cohort	84%	43%

DBT- 5mm: *Duration of the Base of the Triangle at 5 mm from r'-wave;* ***DBT- iso:*** *Duration of the Base of the Triangle at the Isoelectric Line*
